# Child Survival Strategies: Assessment of Knowledge and Practice of Rural Women of Reproductive Age in Cross River State, Nigeria

**DOI:** 10.1155/2016/5098463

**Published:** 2016-10-11

**Authors:** Aniekan Jumbo Etokidem, Ofonime Johnson

**Affiliations:** ^1^Department of Community Medicine, University of Calabar, Calabar 540001, Cross River State, Nigeria; ^2^Department of Community Medicine, University of Uyo, Uyo 520101, Akwa Ibom State, Nigeria

## Abstract

*Introduction*. Nigeria is one of the five countries that account for about 50% of under-five mortality in the world. The objective of this study was to assess the knowledge and practice of child survival strategies among rural community caregivers in Cross River State of Nigeria.* Materials and Methods*. This descriptive cross-sectional survey used a pretested questionnaire to obtain information from 150 women of reproductive age. Data analysis was done using SPSS version 20.* Results*. The child survival strategy known to most of the respondents was oral rehydration therapy as indicated by 98% followed by female education by 73.3% and immunization by 67.3%. Only 20% of the respondents had adequate knowledge of frequency of weighing a child while only 32.7% knew that breastfeeding should be continued even if the child had diarrhea. More respondents with nonformal education (83.3%) practiced exclusive breastfeeding of their last children compared to respondents with primary education (77.3%), secondary education (74.2%), and tertiary education (72.2%).* Conclusion*. Although respondents demonstrated adequate knowledge and practice of most of the strategies, there was evidence of gaps, including myths and misconceptions that could mar efforts towards reducing child morbidity and mortality in the state.

## 1. Introduction

Worldwide, nearly 6.6 million under-five children die yearly, translating to about 18,000 under-five deaths every day [1]. About 50% of under-five child deaths occur in only five countries of the world, namely, India, Nigeria, Democratic Republic of the Congo, Pakistan, and China. Two of these countries, India and Nigeria, account for more than one-third of global under-five mortality, contributing 22 percent and 13 percent, respectively [1]. Sub-Saharan Africa and Southern Asia countries are witnessing an increase in under-five mortality despite a drop from 32 percent in 1990 to 18 percent in 2012 in the rest of the world [1]. Sub-Saharan Africa records the highest rates of under-five child mortality in the world, 98 deaths per 1,000 live births. This figure is 15 times the average for developed countries [1].

The 2007 State of the World's Children Report documented that Nigeria is the country with the 14th highest under-five mortality rate in the world [2]. Seven years after, the 2014 edition of the same report ranked Nigeria as the country with the 9th highest under-five mortality rate in the world, with 124 under-five children dying per 1,000 live births. Incidentally, some African countries fare better; for instance, according to the 2014 report, Libya with an under-five mortality of 15/1000 ranks 116th and Seychelles with 13/1,000 live births ranks 125th. Some of the West African countries that have fared better than Nigeria include Senegal and Ghana with under-five mortality rates of 60/1,000 and 72/1,000 live births and ranking of 43 and 36, respectively [2, 3].

Earlier studies on childhood morbidity and mortality and child survival strategies in Nigeria presented some noteworthy findings. According to Policy Project/Nigeria office, the main causes of infant and child mortality in Nigeria include pneumonia, malaria, diarrhea, undernutrition, and vaccine-preventable diseases [4].

Malaria has been reported as the leading cause of childhood morbidity and mortality in Nigeria, accounting for 25% of infant and 30% of childhood mortality [5, 6]. A study among children in Ilorin, Nigeria, found that pneumonia accounted for 13.3% of all hospital admissions during the study period with a male : female ratio of 1.5 : 1 and 60.5% of the children were infants [7].

Inadequate knowledge and practice of child survival strategies by caregivers, as well as myths and misconceptions, contribute to child morbidity and mortality. A study by Tobin et al. found that 90.2% of respondents indicated that when a child is being weaned from breast milk, the child should continue with breastfeeding when diarrhea occurs with 9.8% indicating that breastfeeding should be discontinued with the onset of diarrhea [8]. In a study in Sokoto, Nigeria, Abiola et al. found that 32.4% of mothers studied believed that “evil eye” was the cause of diarrhea [9].

Worried about the alarming under-five mortality rates in developing countries, the WHO in collaboration with UNICEF and the World Bank, developed a set of evidence-based interventions which, when properly implemented, would reduce under-five mortality. This package of interventions was named child survival strategies. Originally, there were four child survival strategies, namely, growth monitoring, oral rehydration therapy, breastfeeding, and immunization, giving the acronym “GOBI” [10]. With time, some other interventions like family planning, female education, food supplementation, and vitamin A administration were added, giving the acronym GOBIFFFA [11].

Currently, child survival centers around newer strategies such as antenatal care attendance, skilled birth attendance at delivery, and commencement of breastfeeding within one hour of delivery. Others include use of insecticide treated bed nets, management of fever, and treatment of acute respiratory infections, amongst others. According to the NDHS 2013 and the National Bureau of Statistics 2014, the percentage of women who had four or more antenatal clinic visits in Cross River State, 58.7%, was lower than the regional figure of 62.7% but higher than the national figure of 51.1% [12, 13]. Similarly, the percentage of children with fever for whom advice or treatment was sought from a health facility or provider was 21.0% which was lower than the regional figure of 28.2% and the national figure of 31.8% for males and 31.1% for females. However, Cross River State fared better in childhood immunization as the percentage of children aged 12–23 months who received specific vaccines at any time before the survey was higher than the national figures for the three vaccines considered, namely, BCG, DPT3, and OPV3.

## 2. Aim of the Study

The aim of this study was to determine the knowledge and practice of child survival strategies among rural community women in Cross River State, Nigeria.

## 3. Materials and Methods

### 3.1. Study Area

The study was carried out in Cross River State of Nigeria. Cross River State is one of the six states in Nigeria's South-South geopolitical zone. With a population of over 3 million, Cross River State is made up of 18 Local Government Areas which are further grouped into three senatorial districts. The low socioeconomic status of rural community dwellers in Cross River State influences the health-seeking behavior of the women both for themselves and for children in their care.

### 3.2. Study Design

This was a descriptive cross-sectional study.

### 3.3. Sample Size Determination and Sample Selection

The sample size for the study was calculated using Leslie Kish formula:

(1) $$\begin{array}{c} n=\frac{z^{2}pq}{d^{2}}. \end{array}$$

In a study by Tobin et al., 90.2% of respondents were of the opinion that a child being weaned from breast milk should continue to receive the same when he/she has diarrhea [8]. Thus, the proportion with the desired positive attribute was taken as 0.902.

Thus

(2) $$\begin{array}{c} n=\frac{\left(1.96\times 1.96\times 0.902\times 0.098\right)}{0.0025}=135. \end{array}$$

Making provision for 10% nonresponse, the sample size became

(3) $$\begin{array}{c} n=135+13.5=148.5\text{ or approximately, }150. \end{array}$$

Convenience sampling method was used to select 150 rural women. A questionnaire was used to collect data from them. The variables in the questionnaire included sociodemographic variables like age, marital status, occupation, religion, educational status, and ethnic grouping. Other variables included knowledge of the strategies that facilitate child survival and practice of the different child survival strategies. Data obtained from the respondents were analyzed using SPSS software version 20. Both univariate and bivariate analysis were performed. Association between categorical variables was explored using Chi square test.

#### 3.3.1. Ethical Consideration

The data for this study were collected in accordance with the Declaration of Helsinki. Informed consent was obtained from the respondents.

## 4. Results

### 4.1. Sociodemographic Variables

Twenty-six percent of the respondents were aged 45 and above while over 90% were Christians. Nearly 19% of the respondents were civil servants while only 8% were full-time housewives. Sixty-percent of the respondents were married. Fifty-four (36%) respondents had tertiary education. The Efiks constituted majority of the respondents, 77 (51.3%) (Table 1).

### 4.2. Knowledge of Child Survival Strategies and Sources of Information

As shown in Tables 2, 3, and 4, the child survival strategy known to almost all of the respondents was oral rehydration therapy, 147 (98%), followed by female education, 110 (73.3%), and immunization, 101 (67.3%). The commonest source of information regarding child survival strategies indicated by the respondents was health talk in health facility, 113 (75.3%). Only 30 (20%) respondents had adequate knowledge of frequency of weighing a child. One hundred and ten (73.3%) respondents knew that vitamin A prevents blindness among children. The majority of respondents, 114 (76%), knew that breastfeeding should be commenced within the first hour of birth just as the majority, 119 (79.3%), agreed that a child should be given the first milk that the mother expresses after childbirth. Similarly, the majority of respondents, 123 (82%), knew that children should be given salt sugar solution when they have diarrhea. Only 49 (32.7%) respondents knew that breastfeeding should be continued even if the child has diarrhea.

### 4.3. Practice of Child Survival Strategies

As shown in Table 5, sixty-two percent of respondents had attended antenatal clinic in the last pregnancy while 88% indicated that their last children had received all age-appropriate vaccinations. With regard to child spacing, 74% of respondents indicated that the interval between their last two deliveries was at least 2 years while 25% practiced exclusive breastfeeding of their last children.

### 4.4. Some Myths and Misconceptions regarding Certain Aspects of Child Survival

**Figure 1:**
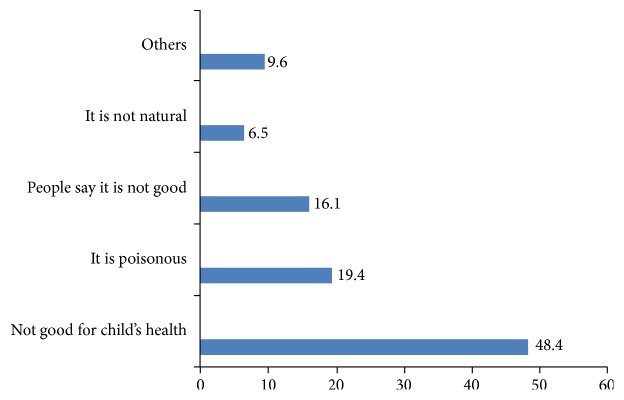
False beliefs about colostrum by respondents.

Figure 1 shows that thirty-one respondents indicated various reasons why colostrum should not be given to the baby; 15 (48.4%) of these indicated that “it is not good for the child's health,” 6 (19.4%) indicated that “it is not natural,” 2 (6.5%) indicated that “it is poisonous,” 5 (16.1%) indicated that “people say it is not good,” and 3 (9.6%) gave various other reasons.

### 4.5. Association between Level of Education and Knowledge of Child Survival Strategies

Table 6 shows that more respondents with tertiary education (57.4%) knew that growth monitoring is a child survival strategy compared with those with secondary education (38.7%), primary education (31.8%), and nonformal education (16.7%). The difference in response between the four categories was statistically significant (Chi square = 9.568, df = 3, *P* = 0.022). Similarly, all respondents with tertiary education (100%) knew that oral rehydration therapy is a child survival strategy compared with 98.4% with secondary education, 95.5% with primary education, and 91.7% with nonformal education. There was no statistically significant difference in the response from the four groups (*P* = 0.1289).

### 4.6. Association between Level of Education and Practice of Child Survival Strategies

Table 7 shows that more respondents with tertiary education (66.7%) attended antenatal clinic in the last pregnancy than those with secondary education, (61.3%), primary education (59.1%), and nonformal education (50%). There was no statistically significant difference between respondents in the four levels of education (Chi square = 1.325; df = 3; *P* = 0.734). Similarly, all respondents with tertiary education (100%) indicated that their last children had received all vaccinations. When this was compared with respondents who had only secondary education (93.5%), primary education (77.3%), and nonformal education (66.7%), there was a statistically significant difference in the response between the four groups (Fisher's Exact Test, df = 3; *P* = 0.001). More respondents with nonformal education (83.3%) practiced exclusive breastfeeding of the last child compared to respondents with primary education (77.3%), secondary education (74.2%), and tertiary education (72.2%). However, there was no statistically significant difference in the response between the four groups (Chi square = 0.733; df = 3; *P* = 0.884). More respondents with tertiary education (87%) were likely to have interval between the last two deliveries equal to or greater than two years compared to those with secondary education (79%), primary education (54.5%), and nonformal education (25%). The difference between the responses from the four groups was statistically significant (Chi square = 24.889; df = 3; *P* = 0.000).

### 4.7. Association between Knowledge and Practice of Child Survival Strategies

As shown in Table 8, children of respondents who indicated that immunization is a child survival strategy were more likely to have been fully immunized than children of those who did not and the difference was statistically significant (*χ* ^2^ = 23.02; df = 1; *P* = 0.0000). There was no statistically significant association between knowledge of the correct definition of family planning and adequate spacing of the last two deliveries.

## 5. Discussion

Only 32.7% of respondents knew that breastfeeding should be continued even when the child has diarrhea. This is comparable to the finding by Tobin et al. that 35.3% of respondents in a related study in a rural community in South-South Nigeria indicated that a child that has diarrhea should be given more breast milk than usual [8]. The findings of these two studies reflect a deficiency in the knowledge and skill of home management of diarrhea among the respondents and are comparable to the finding of a study by Adimora et al. that only 39.4% of mothers of under-five children could correctly manage diarrhea at home while majority (60.6%) could not [14]. The finding of this study is also comparable to that of a related study where Okoh and Alex-Hart found that only 29.3% of respondents had good level of knowledge of home management of diarrhea while only 33.8% had good level of skill [15].

The majority of respondents, 79.3%, agreed that the baby should be given colostrum. This is contrary to the findings of Walia et al. where 66% of respondents withheld colostrums from neonates [16]. In a related study, Morse et al. found that, in 50 out of 120 cultural groups studied, initiation of breastfeeding was delayed by more than 2 days, thus withholding colostrum from the baby [17]. The idea that colostrum is not natural, as indicated by 19.4% of respondents, may be associated with the concept of witch's milk which is the local name given to the milk produced by the breasts of infants. This milk is believed to be abnormal and ancient folklore has blamed it as a source of nourishment for witches' familiar spirits [18]. The close resemblance of colostrum to the so-called witch's milk may explain why the respondents indicate that it is not natural [18]. Regarding reasons why colostrums should not be given to infants, in the study by Walia et al., 54.5% of respondents indicated that it causes obstructions in the intestine while 24.3% indicated that it would be difficult to digest [16].

The proportion of respondents who had antenatal care in the last pregnancy (62%) is comparable to the national average of 61% reported by NDHS 2013 [12]. The high ANC attendance in the state may be due to the free health care for pregnant women and under-five children instituted by the Cross River State Government [19]. Nearly all respondents (98%) knew that ORT is a child survival strategy. This is not surprising because, in Cross River State, the populace is so familiar with salt sugar solution that they even have an apt local name for it, “*mmong uwem,*” which means “life giving water.” This proportion is higher than that of a study by Sanusi and Gbadamosi which found that 78.3% of mothers in Oyo state, Nigeria, practiced ORT as a child survival strategy [20].

Education was found to be a determinant of knowledge and practice of some child survival strategies. It was a determinant of attendance of antenatal clinic although there was no statistically significant difference between the four levels of education (*P* = 0.734). A study by Joshi et al. showed that higher level of education was one of the predictors of both attendance at four or more antenatal visits and receipt of good quality care [21]. Education was a determinant of respondents' children having received all vaccinations, with children of respondents with tertiary education doing better than those of lower levels of education, with a statistically significant difference, *P* = 0.001. Educated women may want to have fewer children that they can cater for (in terms of health and social amenities) and help the children to also be as educated as them.

There was an association between level of education and having received antimalarial medicine in the last pregnancy with a statistically significant difference, *P* = 0.023. These findings compare favorably with those of the NDHS (2013) which documented that women with more than a secondary school education were more likely than other women to have received two or more tetanus toxoid injections during pregnancy. This positive effect of education on the quality of care obtained during antenatal care is further corroborated by another finding by NDHS 2013 that only 43 percent of women with no education used iron tablets or syrup, whereas 74 percent of women with a primary education, 86 percent of women with a secondary education, and 92 percent of women with more than a secondary education did so [12]. The observed inverse relationship between level of education and likelihood of exclusively breastfeeding the last child is likely because women with a higher level of education are more likely than those with lower levels to be employed in jobs that are so demanding that it is difficult for them to exclusively breastfeed their babies. One plausible reason why the breastfeeding prevalence recorded in this study (25%) is higher than the national prevalence (17%) is that 42% of respondents belong to the occupational category of farmers, traders, and full-time housewife. These occupations allow them more time to be within their communities and therefore have access to their babies for breastfeeding.

## 6. Conclusion

Respondents demonstrated adequate knowledge and practice of most of the child survival strategies, especially with regard to oral rehydration therapy. However, there was evidence of some gaps like majority of respondents not knowing that breastfeeding should be continued even when the child has diarrhea. Such inadequate knowledge and practice, including evidence of myths and misconceptions demonstrated by the respondents, could mar efforts to reduce child morbidity and mortality in the state. Education was associated with knowledge and practice of most child survival strategies. This may be an indication that the advocacy for girl child education, which has been intensified within the past 15 years, is gradually making the desired impact and should be encouraged. It is recommended that health care providers should do more to educate caregivers about the basic facts regarding child survival strategies.

## Figures and Tables

**Table 1:** Sociodemographic characteristics.

Variable	Frequency (*n* = 150)	Percent
*Age group*		
15–19	12	8.0
20–24	18	12.0
25–29	18	12.0
30–34	18	12.0
35–39	28	18.7
40–44	17	11.3
45 and above	39	26

*Religion*		
Christianity	142	94.7
Others	8	5.3

*Educational status*		
Nonformal	12	8.0
Primary	22	14.7
Secondary	62	41.3
Tertiary	54	36.0

*Occupation*		
Farming	24	16.0
Trading	27	18.0
Civil servant	29	19.3
Student	24	16
Full-time housewife	12	8.0
Other	34	22.7

**Table 2:** Knowledge of child survival strategies.

Components of child survival strategies correctly indicated	Frequency^*∗*^	Percent
Growth monitoring	64	42.7
Oral rehydration therapy	147	98
Breastfeeding	94	62.7
Immunization	101	67.3
Family planning	65	43.3
Female education	110	73.3
Food supplementation	40	26.7
Essential drugs program	34	22.7
Treatment of common ailments	64	42.7

^*∗*^Multiple responses allowed.

**Table 3:** Source of information.

Source	Frequency^*∗*^	Percent
Health talks in hospital/health center	113	75.3
Personal advice by health worker	77	51.3
Health books	43	28.7
Radio/TV	60	40
Village health worker	41	27.3
Friends and relatives	32	21.3
Patent medicine vendor	7	4.7
Traditional birth attendant	21	14

^*∗*^Multiple responses allowed.

**Table 4:** Adequacy of knowledge of child survival strategies.

Aspect of knowledge	Correct response	
	Frequency (*n* = 150)	Percent
Frequency of weighing of child	30	20
Vitamin that prevents blindness	110	73.3
Food that contains vitamin D	67	44.7
When to commence breastfeeding	114	76
Should a child be given first milk?	119	79.3
Name of the first milk from mother's breast after childbirth	126	84
When should first polio vaccine be given?	105	70
What is exclusive breastfeeding?	117	78
Give salt sugar solution if child has diarrhea	123	82
Continue breastfeeding if child has diarrhea	49	32.7

**Table 5:** Practice of child survival strategies in the last pregnancy and childbirth.

Child survival strategy practiced	Frequency	Percent
Antenatal clinic attendance	93	62
Last child received all immunizations	137	88
At least 2-year interval between last two deliveries	111	74
Exclusively breastfed last child	38	25
Given antimalarial in the last pregnancy	126	84

**Table 6:** Association between level of education and knowledge of child survival strategies.

Independent variable	Dependent variable		Test statistic	df	*P*
	Yes	No			
*Level of education*	*Growth monitoring*				
Nonformal	2 (16.7%)	10 (83.3%)			
Primary	7 (31.8%)	15 (68.2%)	Chi square = 9.568	3	0.022
Secondary	24 (38.7%)	38 (61.3%)			
Tertiary	31 (57.4%)	23 (42.6%)			

*Level of education*	*Oral rehydration therapy*				
Nonformal	11 (91.7%)	1 (8.3%)			
Primary	21 (95.5%)	1 (4.5%)	Fisher's Exact Test	3	0.1289
Secondary	61 (98.4%)	1 (1.6%)			
Tertiary	54 (100%)	0 (0%)			

*Level of education *	*Breastfeeding*				
Nonformal	6 (50%)	6 (50%)			
Primary	12 (54.5%)	10 (45.5%)	Chi square = 2.863	3	0.418
Secondary	38 (61.3%)	24 (38.7%)			
Tertiary	38 (70.4%)	16 (29.6%)			

*Level of education*	*Immunization*				
Nonformal	6 (50%)	6 (50%)			
Primary	12 (54.5%)	10 (45.5%)	Chi square = 7.210	3	0.066
Secondary	40 (64.5%)	22 (35.5%)			
Tertiary	43 (79.6%)	11 (20.4%)			

*Level of education*	*Family planning*				
Nonformal	4 (33.3%)	8 (66.7%)			
Primary	5 (22.7%)	17 (77.3%)	Chi square = 6.659	3	0.084
Secondary	27 (43.5%)	35 (56.5%)			
Tertiary	29 (53.7%)	25 (46.3%)			

**Table 7:** Association between level of education and practice of child survival strategies.

Independent variable	Dependent variable		Test statistic	df	*P*
	Yes	No			
*Level of education*	*Attended antenatal care*				
Nonformal	6 (50%)	6 (50%)			
Primary	13 (59.1%)	9 (40.9%)	Chi square = 1.325	3	0.734
Secondary	38 (61.3%)	24 (38.7%)			
Tertiary	36 (66.7%)	18 (33.3%)			

*Level of education*	*Child received immunizations*				
Nonformal	8 (66.7%)	4 (33.3%)			
Primary	17 (77.3%)	5 (22.7%)	Fisher's Exact Test	3	0.001
Secondary	58 (93.5%)	4 (6.5%)			
Tertiary	54 (100%)	0 (0%)			

*Level of education*	*Interval between deliveries*≥*2 years*				
Nonformal	3 (25%)	9 (75%)			
Primary	12 (54.5%)	10 (45.5%)	Chi square = 24.889	3	0.000
Secondary	49 (79%)	13 (21%)			
Tertiary	47 (87%)	7 (13%)			

*Level of education*	*Exclusive breastfeeding*				
Nonformal	10 (83.3%)	2 (16.7%)			
Primary	17 (77.3%)	5 (22.7%)	Chi square = 0.733	3	0.884
Secondary	46 (74.2%)	16 (25.8%)			
Tertiary	39 (72.2%)	15 (27.8%)			

*Level of education*	*Being given antimalarial medicine during pregnancy*				
Nonformal	7 (58.3%)	5 (41.7%)			
Primary	16 (72.7%)	6 (27.3%)	Fisher's Exact Test	3	0.023
Secondary	55 (88.7%)	7 (11.3%)			
Tertiary	48 (88.9%)	6 (11.1%)			

**Table 8:** Association between knowledge and practice of child survival strategies.

Knowledge variable	Practice variable		df	Chi square	*P* value
*Immunization as a child survival strategy*	*Last child fully immunized*				
	*Yes*	*No*			
Yes	100	1	1	23.02	0.0000
No	37	12			

*Correct definition of exclusive breastfeeding*	*Last child was exclusively breastfed*				
	* Yes*	*No*			
Yes	20	97	1	19.087	0.0000
No	18	15			

*Correct definition of family planning*	*Spacing between last two deliveries is at least two years*				
	*Yes*	*No*			
Yes	31	6	1	2.44	0.1180
No	80	33			
